# Altered cropping pattern and cultural continuation with declined prosperity following abrupt and extreme arid event at ~4,200 yrs BP: Evidence from an Indus archaeological site Khirsara, Gujarat, western India

**DOI:** 10.1371/journal.pone.0185684

**Published:** 2017-10-06

**Authors:** Anil K. Pokharia, Rajesh Agnihotri, Shalini Sharma, Sunil Bajpai, Jitendra Nath, R. N. Kumaran, Bipin Chandra Negi

**Affiliations:** 1 Birbal Sahni Institute of Palaeosciences, Lucknow, Uttar Pradesh, India; 2 Archaeological Survey of India, Vadodara, Gujarat, India; Chinese Academy of Sciences, CHINA

## Abstract

Archaeological sites hold important clues to complex climate-human relationships of the past. Human settlements in the peripheral zone of Indus culture (Gujarat, western India) are of considerable importance in the assessment of past monsoon-human-subsistence-culture relationships and their survival thresholds against climatic stress exerted by abrupt changes. During the mature phase of Harappan culture between ~4,600–3,900yrsBP, the ~4,100±100yrsBP time slice is widely recognized as one of the major, abrupt arid-events imprinted innumerous well-dated palaeo records. However, the veracity of this dry event has not been established from any archaeological site representing the Indus (Harappan) culture, and issues concerning timing, changes in subsistence pattern, and the likely causes of eventual abandonment (collapse) continue to be debated. Here we show a significant change in crop-pattern (from barley-wheat based agriculture to ‘drought-resistant’ millet-based crops) at ~4,200 yrs BP, based on abundant macrobotanical remains and C isotopes of soil organic matter (δ^13^C_SOM_) in an archaeological site at Khirsara, in the Gujarat state of western India. The crop-change appears to be intentional and was likely used as an adaptation measure in response to deteriorated monsoonal conditions. The ceramic and architectural remains of the site indicate that habitation survived and continued after the ~4,200yrsBP dry climatic phase, but with declined economic prosperity. Switching to millet-based crops initially helped inhabitants to avoid immediate collapse due to climatic stresses, but continued aridity and altered cropping pattern led to a decline in prosperity levels of inhabitants and eventual abandonment of the site at the end of the mature Harappan phase.

## Introduction

Archaeological sites in the Kachchh (also called Kutch) region of Gujarat state of western India, located on the western boundary of monsoonal rainfall falling under semi-arid to arid zone with less than ~60 cm annual rainfall [1], represent the northwestern periphery of the Indus (Harappan) civilization, In contrast to the core regions of Indus (Panjab, Haryana and Jammu regions) that receive higher rainfall during the summer (southwest monsoon) and winter (precipitation brought by westerly winds), the Kachchh region is more vulnerable to monsoonal fluctuations and the vagaries of abrupt and extreme climate change. From an archaeological viewpoint, cultural heritage reveals evidences of intimate culture-subsistence-climate relationships in terms of food habits, agriculture, and livestock [2–4]. As the monsoonal rainfall patterns are projected to change rapidly in response to anthropogenically driven warming over large parts of north India [5], it is important to assess the past human response to major phases of monsoonal decline in areas falling under semi-arid to arid zone. Archaeological sites of Gujarat are well suited to examination of climate-culture-subsistence relationships. Available well-dated palaeo records from India, Central Asia, Middle East, and Europe, indicate abrupt and extreme change in climate (monsoon) at ~4,100±100 yrs BP. Increased aridity during this period is considered to be a major factor responsible for collapse of the old kingdom in Egypt, the Akkadian empire in Mesopotamia and Bronze Age societies in Italy, Greece and Crete, and northern Algeria [6–11]. However, there is an ongoing debate regarding the impact of this arid-phase on the Indus (Harappan) Civilization [4, 12, 13].

This debate is centered on whether climate (monsoonal dryness) at ~4,100±100 yrs BP led to a total collapse of the Indus urban settlements or whether these populations survived through adaptation [4–6]. The central question of our investigation is whether changes in subsistence and economic strategies were adopted to maintain cultural continuity following this climatic deterioration. Archaeological sites of western India hold clues to these important issues. Earlier studies [2, 3] suggested that a change in cropping pattern may have been employed as an adaptive measure, but the question still remains as to whether the overall economy and cultural activities of the Harappan inhabitants remained unaffected or showed signatures of fading after ~4,100±100 yrs BP dry-phase of climate?.

**Fig 1:**
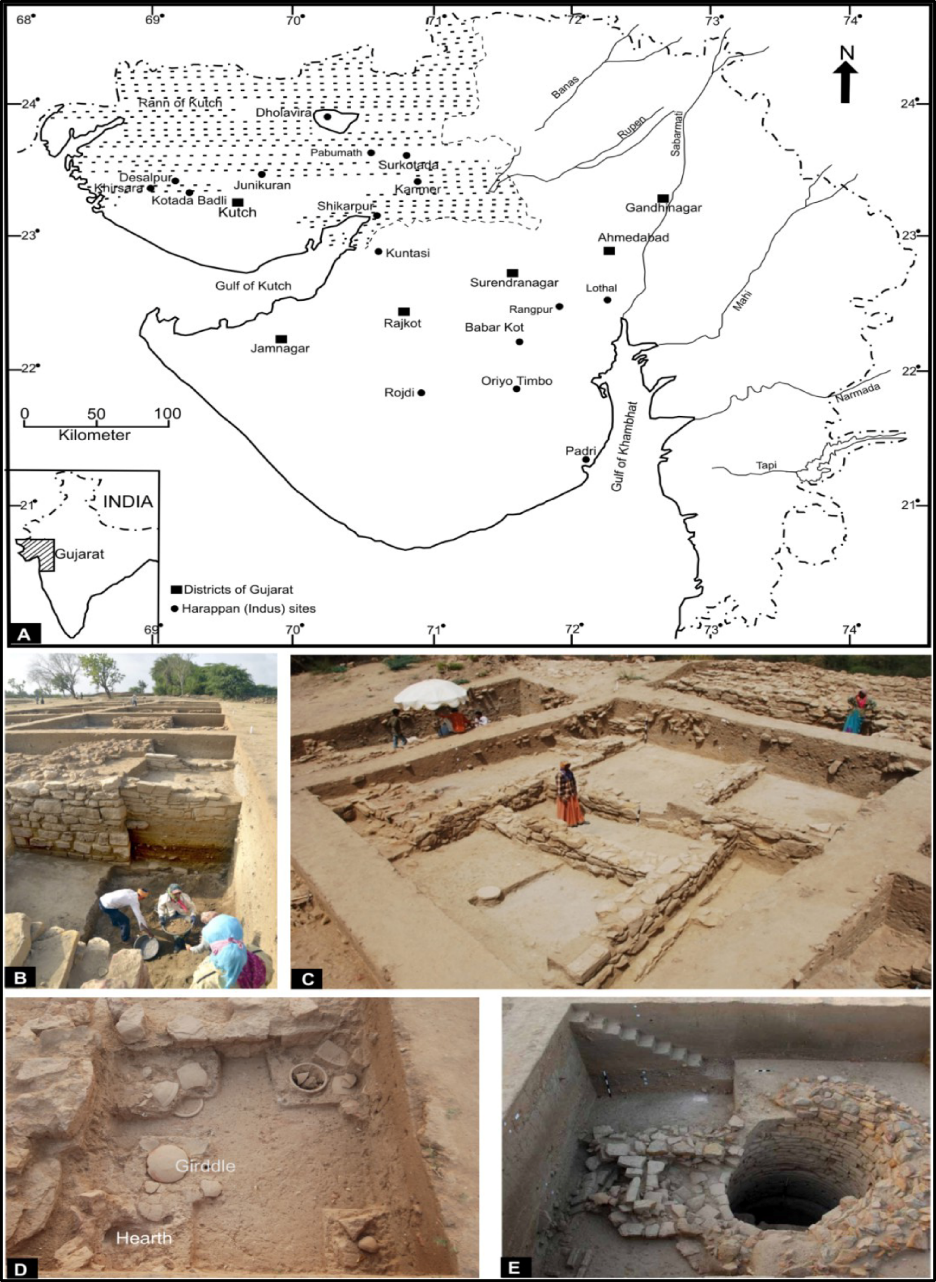
Panel (A) Map of Gujarat showing Khirsara and other Harappan sites discussed in the text. Panel (B, C) General layout of trenches for excavation and excavated trench showing exposed structures and floor level. Panel (D) shows kitchen area with hearth and earthen girdle. Panel (E) shows water well exposed at the site.

Here we present new archaeobotanical, geochemical, and stable isotopic data from an archaeological site Khirsara (23°27′ N; 69°03′ E; Fig 1), where different habitational layers representing the early to late- mature phase of the Indus period have been dated by ^14^C dating of bulk sedimentary organic matter, charcoal, and individual charred agricultural grains. The chief objectives of this study are to evaluate (i) the exact timing of change in cropping pattern at the site (ii) whether these changes were consistently reflected across archaeobotanical, stable isotopic and geochemical datasets and (ii) the efficacy of these changes in maintaining cultural continuity at the archaeological site.

Using a multi-tracer approach employed on sediments of habitational layers at Khirsara, we evaluate regional subsistence pattern of Indus culture in western India vis-à-vis varying monsoonal climate. Data from this early human occupation periodic potentially useful in designing strategies for modern systems dominated by anthropogenically driven global climate variability.

### Geology, climate, and vegetation of the area

The peninsula of Kachchh is marked by low lying bare rocky hills, sandy plains and vast tracts of naked tidal mud flats. The mud flats in the north-western and eastern parts are known as Great Rann and Little Rann, respectively. The Rann consists of tectonic basins bounded by faults, representing filled- up Holocene gulfs [14–17]. The post-glacial Holocene transgression of sea is known to reach its highest mark about ~6000 yrs, when the entire Rann was submerged in seawater and connected with the Gulf of Kachchh and Cambay [18]. The present-day annual rainfall in the Kachchh region is less than ~60 cm, bulk of which is received through southwest monsoon and the remaining in the form of scanty showers during the winter precipitation [1]. According to Champion and Seth’s [19] classification of forest types, the vegetation over Kachchh falls under type 6B/C1 desert thorn forest. Today's vegetation in the vicinity of site mainly comprises *Prosopis juliflora*, *Azadirachta indica*, *Pithecelobium dulce*, *Ziziphus mauritiana*, *Z*. *nummularia*, *Salvadora persica*, *S*. *oleoides*, *Capparis decidua*, *Calotropis* sp., *Ficus* sp., *Mangifera indica*, *Phoenix dactylifera*, *Cocos nucifera* etc.Variety of weeds and wild taxa of family Poaceae, Convolvulaceae,Chenopodiaceae, Asteraceae, and Malvaceae were also noticed in the surrounding area. Due to scarcity of monsoonal precipitation the inhabitants in Kachchh depend largely on summer (kharif) crop that is sown in June-July and harvested in October-November. In areas where irrigation facility has become available in modern times, farmers also cultivate winter (rabi) crops, sown in September-October and harvested in February-March.

### Study site, its historical background, and archeology

The archaeological site (measuring ~310 × 230m) is located ~85 km north-west of Bhuj city of Gujarat, on the south-eastern outskirts of the present day village ‘Khirsara’ overlooking the river Khari. Excavation at the site unearthed the structural remains of a fortified settlement showing planned wall-structures, water-well with a rectangular trough, a citadel, the ware house, factory site and habitational annexes (Fig 1A–1E). The ancient mound locally known as “Gadh Wali Wadi” (fortified area) is located at a height of about ~4.5m above the surrounding area. Excavations were carried out by a team led by Dr. J. Nath, ASI, Vadodara Excavation Branch, Gujarat, during field seasons between 2010 & 2013. The entire site was divided into horizontal grids, each measuring 10m ×10m. The numbers in alphabetical order run north to south and the numerical numbers from west to east. Trenches with Z number such as Z-38 (S1 Fig) and Z-37 are located on higher sections of the mound with a maximum deposit of ~5.6m. The contour map of the excavated site with location of major trenches is shown in S1 Fig.

**Fig 2:**
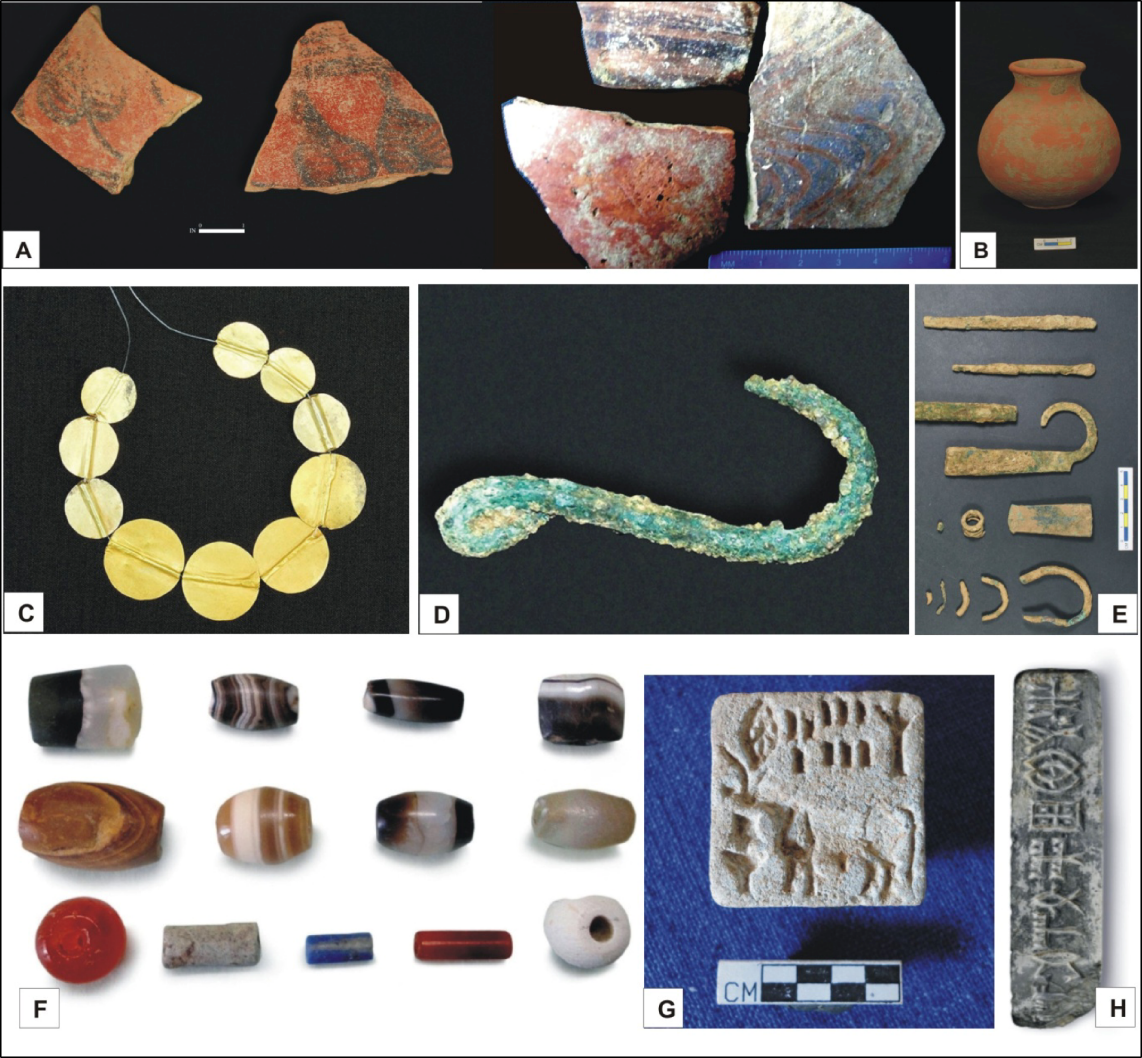
Archaeological artifacts recovered from Khirsara (A) Mature Harappan ceramics (painted and plain) (B) Red Ware Harappan pot (C) Gold beads (D) Fish hook made of copper (E) Copper objects (F) Beads of semiprecious stones (G) Seal with bison (H) Seal bearing Harappan characters. These antiquities have been stored at the office of Excavation Branch-V, ASI, Vadodara, Gujarat, India.

Historically, investigations of the Harappan archaeology began in Gujarat state in 1934–35, when M.S. Vats excavated the first Harappan site at Rangpur in Limdi Taluka, Surendranagar District of Saurashtra region [20]. The archaeological site of Khirsara was first reported by the Department of Archaeology, Govt. of Gujarat in 1969–70. Excavations at Khirsara indicate that the area was likely a habitational-cum-industrial hub manufacturing potteries, shell bangles, beads etc. Discovery of the shell-working areas, inlay hoards and abundant copper objects (*e*.*g*. beads, spacers, bangles, rings, knives, chisels, nails, needles, arrow heads, fish hooks) revealed contemporary trade activities [21]. Also discovered at this site were Harappan artifacts such as steatite and semiprecious stone beads, chert-blades, sling balls, shell bangles pieces etc. and a variety of pottery including perforated jar, dish-on-stand, goblets, and Reserve Slip ware sherds with painted motifs [22]. A variety of typical Harappan pottery both plain and painted type predominantly consisted of Red Ware. Beads of gold, copper objects, semi-precious stones like carnelian, agate, chert, chalcedomy, jasper, lapis-lazuli as well as faience, steatite shell and terracotta have been found in large numbers (Fig 2A–2F). Seals of square, rectangular and bar type made of steatite, soap stone and sandstone have also been recovered from Khirsara (Fig 2G–2H), besides some typical Harappan legends, the rectangular seals representing figurines of unicorn and hump-less bull on the obverse. The reverse side of every rectangular seal has a knob with perforation, whereas the bar type seal bears a perforated bass at the back [21]. Overall, the artifact assemblage indicates a prosperous socio-economic status, possibly supported by optimal climatic conditions. Other contemporary Harappan excavations at Rangpur, Pabumath, Lothal, Shikarpur, Rojdi, Oriyo Timbo, Surkotada, Padri, Kuntasi, Dholavira, Bagsara, Juni Kuran, Kanmer and Kotada-Badli [23–32], revealed similar prosperity and technical sophistication in town planning and utilization efficacy of various available resources.

Archaeological finds from Khirsara indicate that the entire deposit belongs to a Mature Harappan phase which can be primarily divided into two sub-phases (A and B) [21]. Evidence of a protection-wall running almost parallel (~2.35 m wide) to the fortification-wall was noticed at a distance of 5.8m on the north and ~20m in the east. This indicates that the ancient inhabitants were likely concerned about the probable flood related threats from the river Khari which flows ~300m away from the mound.

## Material and methods

### Sampling of macrobotanical remains and their identification

Botanical remains were retrieved using water-floatation technique that relies on density differences of organic and inorganic material enhancing quantity of the botanical material in the floated portion. Soil from the cultural deposits, pits, floor and hearth was poured into the plastic tub filled with water, and the material was agitated, so that light botanical material is buoyed to the surface. The floating material was skimmed off through a 0.5mm sieve and collected in a cloth, tagged, and allowed to dry in shade. After drying the material was packed in polythene bags, with archaeological provenience for laboratory study.

**Fig 3:**
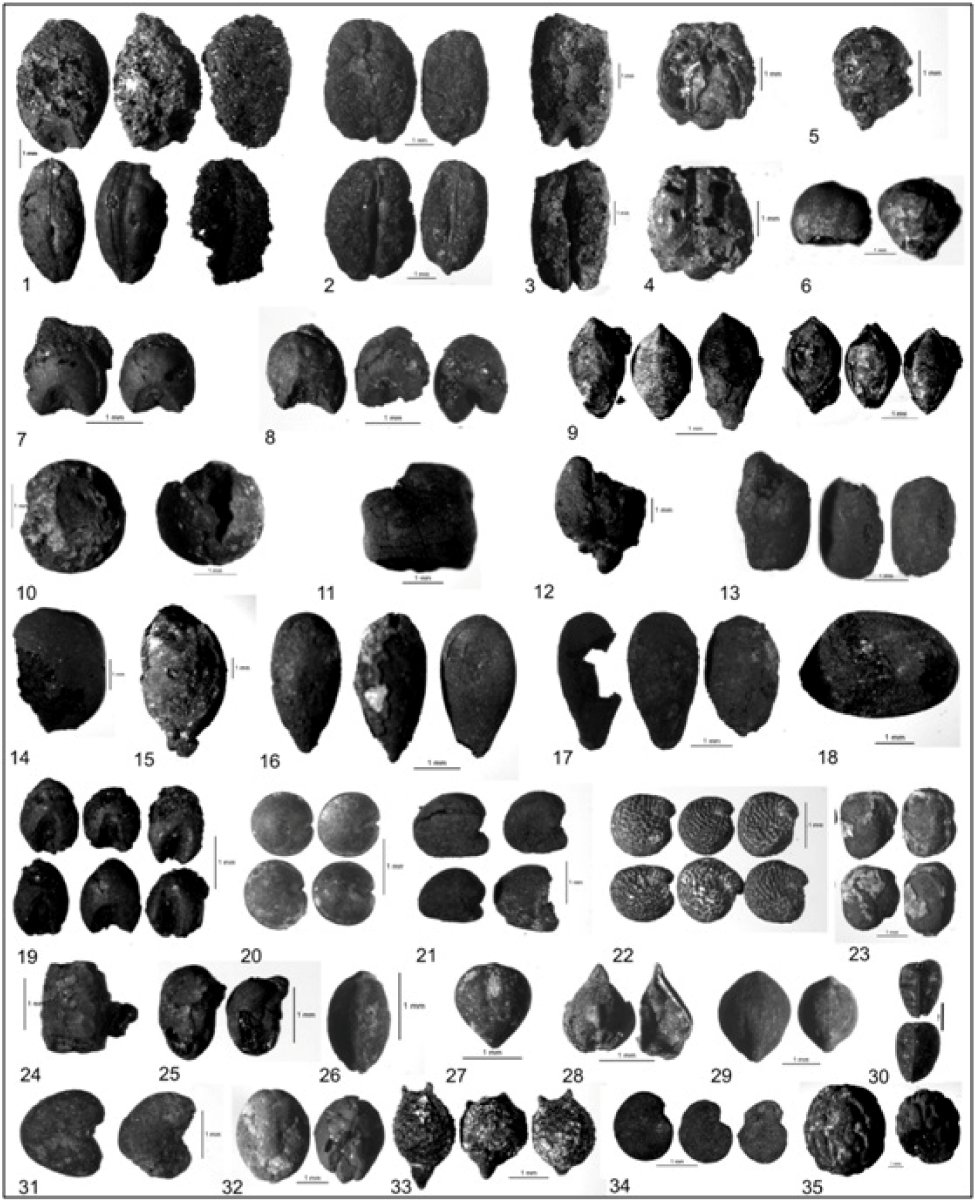
Macroscopic plants remains of cultivars, weeds and wild taxa (1) *Hordeum vulgare* (2) *Triticum aestivum/durum* (3) *T*. cf. *dicoccum* (4) *T*. cf. *sphaerococcum* (5) *Pennisetum glaucum* (6) *Sorghum* cf. *bicolor* (7) cf. *Eleusine coracana* (8) *Panicum* cf. *miliaceum* (9) *Setaria* cf. *italica* (10) *Pisum* cf. *arvense* (11) *Lathyrus sativus* (12) *Cicer* cf. *arietinum* (13) *Vigna* cf. *radiata* (14) *Macrotyloma uniflorum* (15) cf. *Luffa* sp. (16) *Linum* cf. *usitatissimum* (17) *Sesamum indicum* (18) *Gossypium arboreum/herbaceum* (19) *Setaria* sp.(20) *Celosia* sp. (21) *Sida* sp. (22) *Trianthema* cf. *triquetra* (23) *Indigofera* sp. (24) *Indigofera* cf. *hirsuta* (25) *Trigonella* cf. *occulta* (26) *Cyperus* sp. (27) *Scirpus* sp. (28) *Rumex* sp. (29) *Polygonum* sp. (30)*Asphodelus* sp. (31) *Abutilon* sp. (32) *Ipomoea* sp. (33) *Scleria* sp. (34) *Solanum* sp. (35) *Ziziphus* cf. *nummularia*. *All the macrobotanical remains are stored in the Museum of Birbal Sahni Institute of Palaeosciences*, *Lucknow*, *U*.*P*., *India (BSIP St*. *no*. *1439)*.

The samples comprising carbonized botanical remains were examined under stereo-binocular microscope (Leica Z6APO®). Grains, seeds, and fruits were then separated out and kept in vials for photo-documentation and measurements. Botanical remains were found in utterly fragile, highly burnt and damaged state of preservation. The Identification of carbonized grains/seeds is based on morphological details preserved in them [33, 34] (Fig 3). A total of 1269 plant remains of both cultivated and wild species were recovered from 69 samples, collected through floatation of ~6600 litre of sediment randomly from all over the site. Absolute count of plant taxa, ubiquity and diversity index (Shannon-Weaver Index) are given in Table 1.

**Table 1:** Abundance, ubiquity and diversity index of charred remains from occupational phases at Khirsara (4600–3900 yrs BP).

	4600–4300 yrs BP: 1A (17 samples)			4300–4100 yrs BP: TP (9 samples)			4100–3900 yrs BP: 1B (43 samples)			Diversity Index			
TAXA	Absolute counts	present in samples	Ubiquity	Absolute counts	present in samples	Ubiquity	Absolute counts	present in samples	Ubiquity	Total	1A	TP	1B
*Hordeum vulgare*	44	11	64.7	18	6	66.7	38	19	44.2	100	0.16	0.12	0.05
*Triticum* cf. *aestivum*	0	0	0.0	2	1	11.1	1	1	2.3	3	0.00	0.03	0.00
*Triticum* cf. *dicoccum*	0	0	0.0	1	1	11.1	1	1	2.3	2	0.00	0.02	0.00
*Triticum* cf. *sphaerococcum*	0	0	0.0	0	0	0.0	1	1	2.3	1	0.00	0.00	0.00
cf. *Eleusine coracana*	0	0	0.0	2	1	11.1	11	4	9.3	13	0.00	0.03	0.02
*Pennisetum glaucum*	0	0	0.0	0	0	0.0	2	2	4.7	2	0.00	0.00	0.01
*Sorghum* cf. *bicolour*	0	0	0.0	1	1	11.1	2	1	2.3	3	0.00	0.02	0.01
*Panicum miliaceum*	0	0	0.0	0	0	0.0	7	2	4.7	7	0.00	0.00	0.01
*Setaria* cf. *italic*	0	0	0.0	0	0	0.0	31	2	4.7	31	0.00	0.00	0.05
*Pisum* cf. *arvense*	1	1	5.9	0	0	0.0	0	0	0.0	1	0.02	0.00	0.00
*Cicer* cf. *arietinum*	0	0	0.0	1	1	11.1	0	0	0.0	1	0.00	0.02	0.00
*Lathyrus sativus*	1	1	5.9	0	0	0.0	0	0	0.0	1	0.02	0.00	0.00
*Macrotyloma uniflorum*	2	1	5.9	1	1	11.1	3	3	7.0	6	0.03	0.02	0.01
*Vigna* cf. *radiate*	2	2	11.8	1	1	11.1	2	1	2.3	5	0.03	0.02	0.01
*Linum* cf. *usitatissimum*	2	1	5.9	2	1	11.1	33	6	14.0	37	0.03	0.03	0.05
*Sesamum indicum*	0	0	0.0	0	0	0.0	37	8	18.6	37	0.00	0.00	0.05
*Gossypium arboreum/herbaceum*	0	0	0.0	0	0	0.0	3	2	4.7	3	0.00	0.00	0.01
*Setaria* sp.	8	5	29.4	55	5	55.6	625	22	51.2	688	0.08	0.16	0.13
*Polygonum* sp.	0	0	0.0	0	0	0.0	2	2	4.7	2	0.00	0.00	0.01
*Rumex* sp.	1	1	5.9	0	0	0.0	1	1	2.3	2	0.02	0.00	0.00
*Scirpus* sp.	0	0	0.0	0	0	0.0	1	1	2.3	1	0.00	0.00	0.00
*Cyperus* sp.	1	1	5.9	0	0	0.0	0	0	0.0	1	0.02	0.00	0.00
*Ipomoea* sp.	2	1	5.9	0	0	0.0	6	3	7.0	8	0.03	0.00	0.01
*Celosia* sp.	2	1	5.9	1	1	11.1	1	1	2.3	4	0.03	0.02	0.00
*Asphodelus* sp.	0	0	0.0	0	0	0.0	15	4	9.3	15	0.00	0.00	0.03
*Sida* sp.	0	0	0.0	0	0	0.0	3	2	4.7	3	0.00	0.00	0.01
*Zaleya* sp.	33	0	0.0	26	6	66.7	104	24	55.8	163	0.16	0.14	0.10
*Ziziphus* sp.	14	5	29.4	12	4	44.4	20	11	25.6	46	0.11	0.10	0.03
*Scleria* sp.	0	0	0.0	0	0	0.0	44	6	14.0	44	0.00	0.00	0.06
*Tradescantia* sp.	0	0	0.0	0	0	0.0	0	0	0.0	0	0.00	0.00	0.00
*Trigonella* cf. *occulta*	0	0	0.0	0	0	0.0	0	0	0.0	0	0.00	0.00	0.00
*Indigofera* sp.	0	0	0.0	0	0	0.0	35	3	7.0	35	0.00	0.00	0.05
*Abutilon* sp.	0	0	0.0	0	0	0.0	3	2	4.7	3	0.00	0.00	0.01
Cucurbitaceae type (*Luffa*)	0	0	0.0	0	0	0.0	1	1	2.3	1	0.00	0.00	0.00
**Total**	**113**			**123**			**1033**			**1269**	**0.74**	**0.71**	**0.72**

### Radiocarbon dating

The absolute chronology of the archaeological site is based on dating of different habitational layers from multiple trenches. Suitable habitational/ cultural layers were identified from different trenches based on their archaeological finds and geological settings. ^14^C dates measured by both AMS and conventional beta counting (on Liquid scintillation counter; LSC) of bulk sedimentary organic matter, charcoal were used to ascertain chronology of different depths. The LSC radiometric dating was carried out at C-14 laboratory of Birbal Sahni Institute of Palaeosciences(BSIP), Lucknow, India (Table 2), whereas AMS dating of three sediment-layers from the trench AF-35 (used for C isotopic studies) was carried out at Institute of Physics, Gliwice Radiocarbon Laboratory (GdA), Poland (Table 3). AMS measured ^14^C dates of agricultural grains (Barley and Wheat) from the trench Z-38 were obtained from a radiocarbon facility Beta-Analytica (USA) (Table 4).

**Table 2:** ^14^C dates of wood-charcoal measured by conventional beta counting method at BSIP.

Trench	Depth (cm)	Lab Ref. No. BSIP, Lucknow	^14^C date (yrs BP)	Calibrated Age (BC) (with 2σuncertainty i.e. 95.4% confidence level)	Calibrated Age (BP) (with 2σ uncertainty i.e. 95.4% confidence level)
**Z-38/1**	530–560	BS-3362	3900±70	2198–2505	4147–4454
**Z-38/1**	500–550	BS-3358	3860±70	2136–2492	4085–4441
**Z-38/1**	450–460	BS-3310	3920±90	2137–2666	4086–4615
**AE-35/4**	221–251	BS-3471	3290±70	1428–1700	3377–3649
**AE-35/4**	210–240	BS-3469	3390±70	1527–1882	3476–3831
**Z-37/2**	205–215	BS-3354	3390±70	1527–1882	3476–3831
**S-37/1**	200–210	BS-3468	3800±80	2026–2471	3975–4420

**Table 3:** AMS measured ^14^C dates of soil-sediment from the trench AF-35. The first sample* was dated with conventional Beta counting method at BSIP.

Trench	Depth (cm)	Lab Ref. No.	^14^C date (yrs BP)	Calibrated Age (BC) (with 2σ uncertainty i.e. 95.4% confidence level)	Calibrated Age (BP) (with 2σ uncertainty i.e. 95.4% confidence level)
*AF-35/1	190	BS-3466	3230±70	1386–1685	3335–3510
**AF-35/1**	220	GdA-2850	3810±25	2195–2340	4144–4289
**AF-35/1**	275	GdA-2849	3805±25	2143–2309	4092–4258
**AF-35/1**	400	GdA-2847	4155±25	2659–2876	4608–4825

**Table 4:** AMS measured ^14^C dates of agricultural grains (Barley and Wheat respectively) from the trench Z-38.

Trench	Depth (cm)	Lab Ref. No.	^14^C date (yrs BP)	Calibrated Age (BC) (with 2σ uncertainty i.e. 95.4% confidence level)	Calibrated Age (BP) (with 2σ uncertainty i.e. 95.4% confidence level)
*Z-38/1	530–560	Beta-427231	3750±30	2038–2231	3987–4180
**Z-38/1**	530–560	Beta-422014	3760±30	2091–2287	3993–4236

Final ^14^C ages were determined from the ^14^C/^12^C ratios after normalizing carbon isotopic fractionation measured by δ^13^C = –25.0‰ [35] and then calibrated to assess calendar ages. Calibration was conducted with the probability method of OxCal v 4.1.7 [36] and the IntCal13 data set [37]. The age ranges presented in Tables 2, 3 and 4 represent the maximum probability at 95.4% confidence level on calendar year scale.

### Stable carbon isotopic measurements

For carbon isotope (δ^13^C) analyses, a total of thirty sediment samples were used. All samples were taken from a trench AF-35, excavated to a depth of ~4.55 m with the help of a trowel at 15 cm intervals. The trench showed conspicuous stratified layers (without any mixing or overlapping). Soil sediment samples were decalcified using 5% Hydrochloric acid (HCl) overnight for 8–10 hours, washed several times with deionized water and dried in an oven at ~50°C. After drying, the sediment samples were mildly re-powdered in agate mortar and transferred into dried clean plastic vials. For mass-spectrometric analysis, aliquots ranging from ~20–40 mg of dried powder were weighed in clean tin cups and sealed into pellets by nicely and gently pushing from all sides to remove any entrapped gases. All samples were packed in oval shaped pellets. Using auto sampler, these pellets were dropped into the reactor of elemental analyzer (1112 Flash EA; Thermo®) interfaced with a MAT253 stable isotope mass-spectrometer in a continuous flow mode at Birbal Sahni Institute of Palaeosciences, Lucknow, India. Accuracy and precision of δ^13^C data were found to be better than 0.1‰ and 0.08‰, respectively. The isotopic data are reported using the standard delta notation with respect to Vienna-PDB. Quality of measured δ^13^C data was checked using a suite of in-house and international standards.

## Results and discussion

^14^C dates from all trenches and associated ceramic material suggested that this site can be divided into two phases: the early Phase IA (~4600–4300 yrs BP), and the mature Phase IB (~4100–3900 yrs BP) separated by a Transitional Phase (TP) (~4300–4100 yrs BP). The entire sequence corresponds mainly to the mature phase of Harappan cultural period. Macrobotanical remains, stable C isotopic data, and the ceramic data belonging to these different phases are discussed below.

### Macrobotanical remains

**Fig 4:**
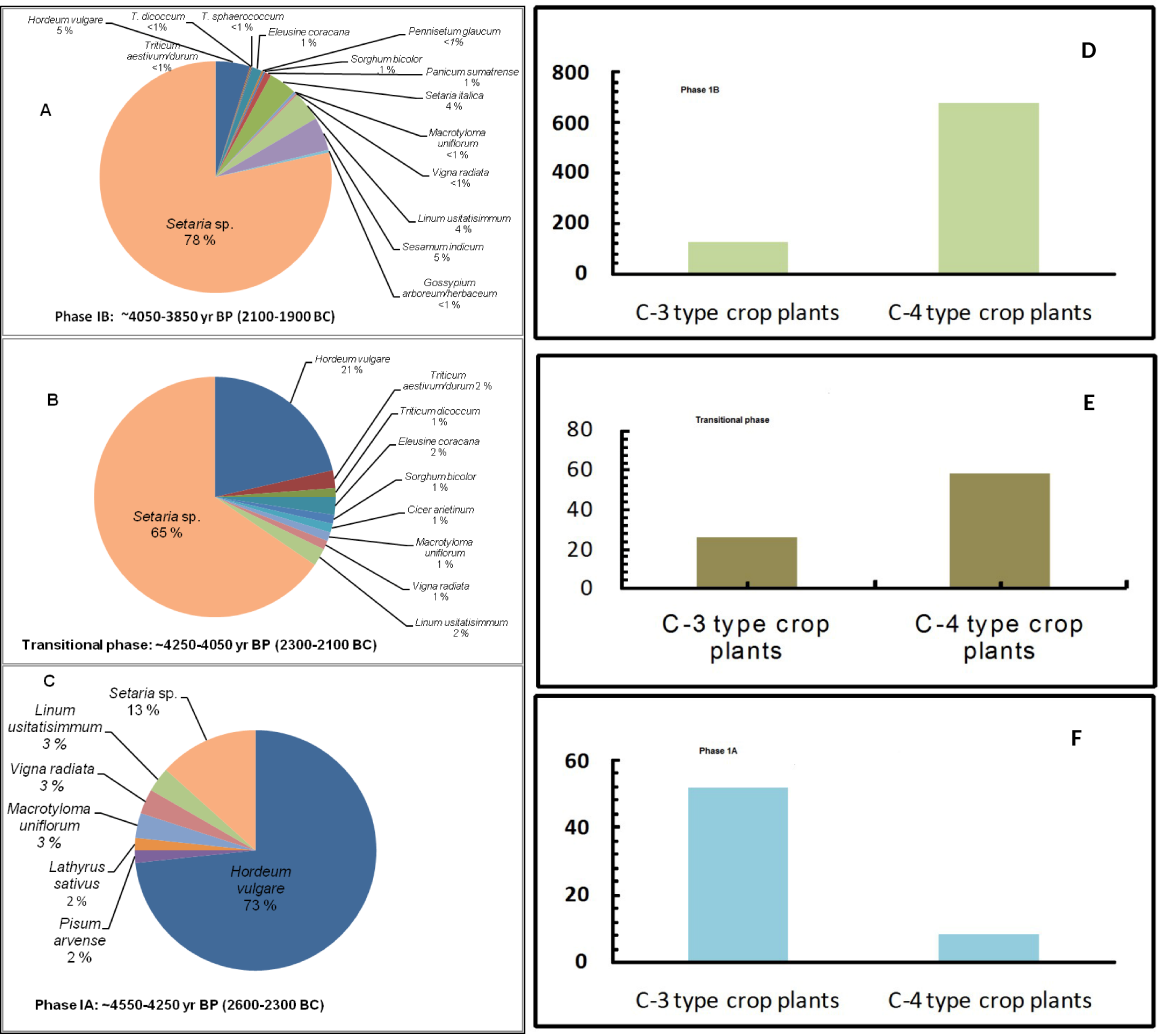
Left panels show pie-charts displaying relative abundance of crop plants and right panels show corresponding histograms for three distinct phases of overall subsistence changes in the archaeological site Khirsara.

The carbonized remains show contemporary crop economy and ecology of the area between ~4600 to ~3900 yrs BP. Plant remains of cultivars, weeds, and wild-taxa, generally indicate a double-cropping pattern. Segregated crop remains were quantified based on the total number of grains/seeds of a particular species over total number of crop species from three distinct temporal phases (1A, TP, and 1B; Fig 4). These phases were characterized by distinct ceramic types in the habitational layers (discussed in a later section). Statistical validation of abundance of macrobotanical remains was undertaken to correlate changes in crop-assemblage with the cultural phases. Distinctness of Identified phases was ascertained using *Kruskal-Wallis* statistical test (by rejecting null hypothesis) and non-parametrical ANOVA (one way) test using SPSS software (version# 21). The three phases were found to be distinctly different at 95% confidence level (p = 0.05).

Phase 1A (Fig 4C; ~4600–4300 yrs BP) demonstrates a greater range of crop plants. A diversity index (0.74) during this phase shows that a large number of plant taxa are evenly distributed (Table 1).The winter crops (barley-wheat, field pea, grass pea, linseed) accounted for ~80%, whereas the summer crops (such as green/ yellow/ bristley, horsegram and green gram) accounted for ~20%, indicating availability of sufficient moisture due to winter and summer precipitation. This inference was corroborated by sediment-core studies conducted in western India [38–40]. Amongst crops, the large grained cereal (barley) appears to be the main crop which accounted for ~73%, whereas the small-grained millets were low in abundance accounting for ~14%.

During the transitional phase (~4300–4100 yrs BP), the double-cropping pattern appears to continue; however, the abundance of large grained cereals (barley, wheat) declined significantly, accounting for only ~24% in contrast to small-grained millets (green/yellow/bristly, foxtail, sorghum, finger millet (ragi) representing ~68% of the total agriculture produce (Fig 4B). In the later phase of the mature Indus cultural era i.e. phase 1B (~4100–3900 yrs BP; Fig 4A), the major crops (large-grained cereals) declined significantly, whereas small-grained millets (green/yellow bristly foxtail, foxtail, proso millet, sorghum, pearl millet, finger millet (ragi)) dominated the agricultural produce with ~85% abundance (Fig 4A).The abundance of *Setaria* sp. grains suggest that they did not arrive just as contaminants but were gathered from the crop harvested. The change in abundance pattern of dominating crops is shown both by pie-chart as well as through histogram representation (Fig 4). The strategic shift towards monsoon-suitable crops might be due to human adaptation during prolonged drought conditions. Similar evidence of agricultural shift has also been recorded for the late mature phase (4,100–3,900yrs BP) at Kanmer [2,3] and Kotada-Badli (~4,200–3,900yrs BP [Pokharia, unpublished data] in the same ecological zone. The typical cultivated foxtail millet of Chinese origin has been reported since ~4,500 yrs BP in the subcontinent [41, 42].

### Carbon isotopes (δ^13^C) of organic matter

**Fig 5:**
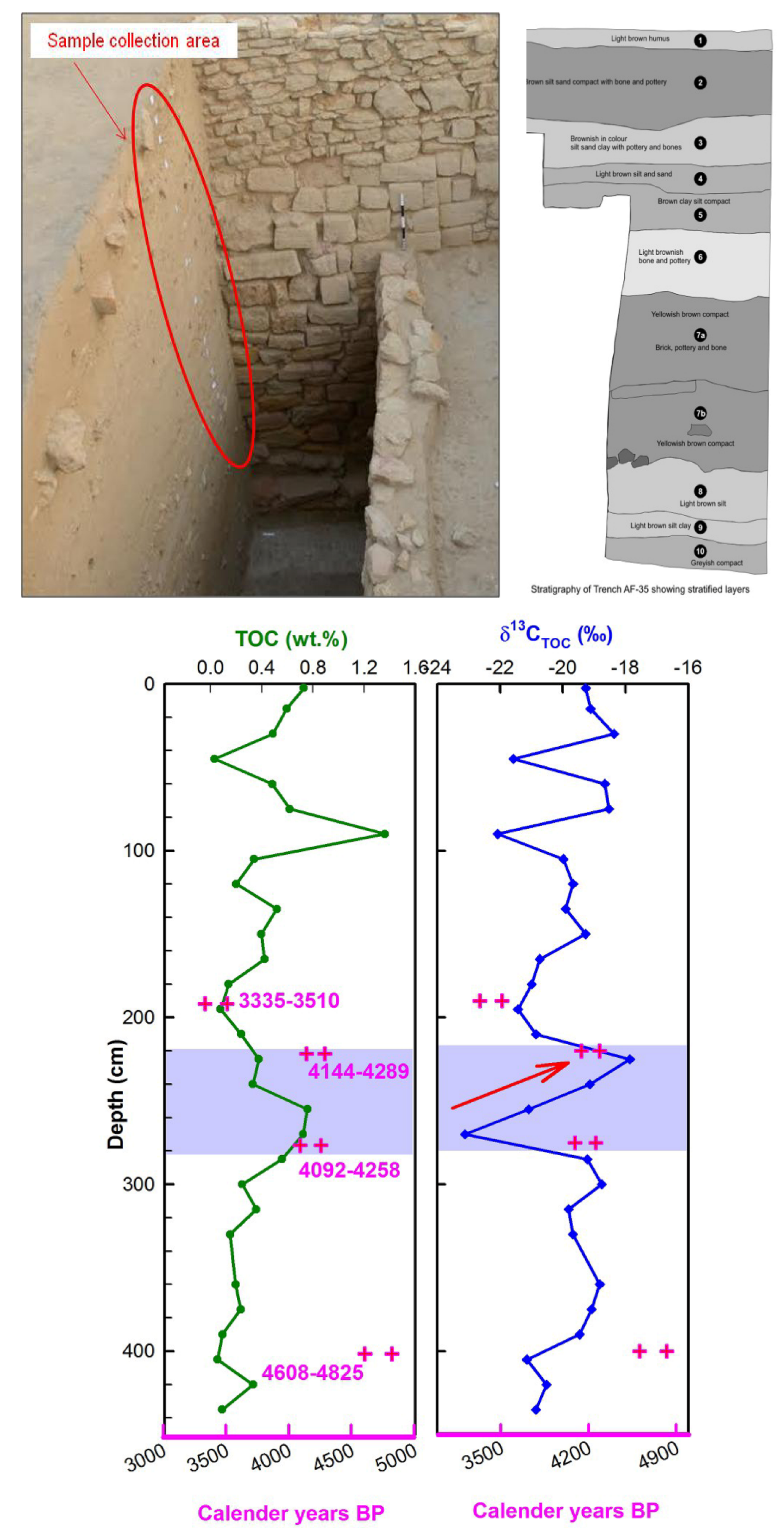
Photograph of trench AF-35 from where samples were taken for C isotope analysis. Right panel shows stratigraphic appearances with finds of archaeological artefacts. Depth-profiles of TOC (wt.%) and δ^13^C_TOC_ of the trench AF-35 is shown in lower panels. Calibrated ^14^C age based chronological constraints are shown as + symbols.

The soil organic carbon TOC (wt.%) and its carbon isotopic anomaly (δ^13^C_SOM_) measured from the AF-35 trench is shown in Fig 5. Age control of the trench is based on four ^14^C dates, three of these being AMS^14^C calibrated ages (Table 3) and the fourth one being a conventional ^14^C age (Table 2) (Fig 5). Depth-variations of δ^13^C_SOM_ values show a steep rise of ~5.2‰ between 2.25–2.70 m depth-interval which yields two overlapping AMS ^14^C ages dating right at ~4200 yrs BP. This time-slice is well known as a wide-spread aridity epoch lasting probably two to three hundred years [12,13,43].

δ^13^C_SOM_ is known to represent the δ^13^C of bulk vegetation which could be C3, C4 or CAM type [44]. Other environmental factors may also influence δ^13^C_SOM,_ such as (i) decomposition effects of SOM (enriching δ^13^C), (ii) precipitation/ aridity effects (δ^13^C ofC3 plants increases with decreasing precipitation/ increasing aridity) and (iii) changes in atmospheric C isotope ratio i.e. δ^13^C of CO_2_ used in photosynthesis. These plausible environmental influences need to be evaluated in the context of soil profile and the period of deposition. The first of these possibilities i.e. decomposition effects could be significant in natural ecosystems for longer durations (at least decadal to millennial timescales), but this possibility is highly unlikely for the Khirsara site and cannot account for a massive change in δ^13^C_SOM_ seen at ~4200 yr BP_._ The second possibility i.e. increased aridity in the region can enhance δ^13^C_SOM_ (without change in crop-type) but this possibility is unlikely as the enrichment in δ^13^C ofC3 plants with decreasing precipitation is generally in order of ~0.001‰/ mm, hence unlikely to account for a massive ~5.2‰ change. The third possibility is also out of question since δ^13^C of atmospheric CO_2_ has remained fairly constant (within ~0.2‰) during the entire Holocene epoch.

As the studied section AF-35 δ^13^C_SOM_ is from Khirsara archaeological site which was habitational, the change in δ^13^C_SOM_ is most likely due to crop change. The barley-wheat based crops are C3 type with typical δ^13^C values ~ –23±1‰ and distinctly different from millet-based (C4 type) crops with typical δ^13^C values ~ ‒8±1‰ [44]. Since the macrobotanical assemblage shows significant crop-pattern changes at ~4200 yrs BP, we argue that the observed change in δ^13^C_SOM_ at 4200 yrs BP (Fig 5) is also most likely due to change in crop-type (from C3 type barley-wheat based crops to C4 type millet based crops). Prevailing aridity at ~4200 yrs BP most likely forced cultivators to opt for C4 type millet- based crops as they require less water to grow. Furthermore, the studied AF-35 section represents a habitational repository, not a natural soil-profile. Hence, δ^13^C_SOM_ change at ~4200 yr BP (Fig 5) can be best explained in terms of change in the nature of abundance of food grains (from barley-based to millet-based crops) rather than vegetation type [42].

In the upper section of Fig 5, δ^13^C_SOM_ again fluctuates between ~100–50 cm depths. However, in absence of data on radiocarbon ages and the abundance pattern of macrobotanical remains from this level (Fig 4D), it is difficult to offer a viable interpretation. As significant crop change to C-4 type (millet-based crops) had already been adopted, a change in subsistence pattern yet again seems unlikely. It has to be noted that the Khirsara archaeological site falls under the semi-arid to arid zone of Indian monsoon coverage area [1], where sharp changes in summer-monsoon intensity can severely impact local agronomy, crop-cultivation and crop-yield. Dry phases of climate in the northwestern India have been shown to favor millet-based crops [2, 3]. The occurrence of significant change in crop-type from barley-based to millet-based crops at ~4200yrs BP from a well-dated archaeological site falling under semi-arid zone of Gujarat is noteworthy in context of some recent reports from the core region of Indus culture that push the times of arrival and demise of Harappan culture back in time [4].

The consistency of ^14^C ages of organic matter contained in soil sediment and that of botanical remains (seeds/ grains) is excellent, as suggested by overlapping^14^C dates for the depth interval 530–560 cm of trench Z-38 (Tables 2 and 4). This observation further supports our interpretation of δ^13^C_SOM_ of trench AF-35 in terms of prevailing changes in crop-type.

### Architectural and ceramic data

The architectural and ceramic data from any archaeological site are of great importance in our understanding of social and economic complexity and subsistence strategies. At Khirsara, the ceramic and architecture remains provide evidence for two distinct phases (1A and 1B), with the indistinct transitional phase (TP) possibly being too small to be reflected in the ceramic data. The entire sequence can be sub-divided into a mature (developed; 1A) and late-mature (declining; 1B) phases with an intermittent TP. The phase 1A showed three structural phases, whereas phase 1B revealed two structural phases. The structures belonging to phase 1A were raised with sandstone with mud-mortar and multi-color mud bricks, frequently used in the citadel area. It appears that each sub-phase was destroyed by the flood events and that the resident humans overcame this problem by raising the height of the structure. During the phase 1B (the declining phase after ~4200 yrs BP aridity event), the structures were mostly of single- or double-course, raised by leveling the phase 1A structures.

The ceramic wares of phase 1A are of medium thickness, smooth, and well fired. Ceramics found in this phase were mainly red ware, red slip ware, buff ware, buff slip ware, red ware, gritty red ware, black ware, grey ware, reserve slip ware and perforated ware. Uniformly burnt sections of vessels indicate the use of high temperature under controlled conditions as revealed by kilns of varied shapes.

In contrast, during phase 1B, the ceramic types were of coarser variety, including coarse red ware, coarse black ware, micacious red ware, coarse grey ware, and reserve slip ware. The coarser wares were found to be poorly fired with rough surfaces. This suggests a likely decline in prosperity at the site, plausibly owing to prevalent arid conditions. This effect could also be noticed in the poorer fabric, in the carelessness shown in painting the vessels, and in the gradual disappearance of elaborated forms such as goblets, beakers, buff ware and perforated sherds. Hence, we surmise that changes in food habits after the dry phase might have affected the socio-economic conditions significantly.

### Culture-subsistence-climate relationship

The combined culture-subsistence-climate database recovered from the archaeological site at Khirsara is important for the insights it provides in to past human civilization living in a monsoonal drought-prone semi-arid area of western India. Khirsara archaeological site lies on the trade route to Sindh (now in Pakistan) and is situated on the outer edge of Khirsara village, with saucer-like shape, with mounds on all sides and a depression in the middle. Massive structures, fortification, citadel complex, industrial area, warehouse, water reservoir, pottery kiln (rock cut), pottery, seals, weights, terracotta cakes, beads of semi-precious stones and gold, copper implements, shell bangles etc. are features that indicate that the site belonged to a mature Harappan phase which continued to flourish for ~700 years from ca. ~4600 to ~3900yrs BP. Seals found at the site also range from early to the late stage of the mature Harappan phase.

The favorable climatic conditions with sufficient precipitation during both ‘summer and winter likely stimulated agricultural activities initially and supported urbanization around ~4,600yrs BP (~2600 BC). A recent study [2] from the peripheral zone of Indus civilization (Kanmer 23°23′N; 70°52′E; belonging to Early-Mature Harappan time period) also revealed similar subsistence system based on winter and summer crops. Archaeobotanical studies in the core region of the Indus (Harappan) civilization have also shown double cropping pattern during Early and Mature phases [45, 46]. The present evidence from Khirsara site clearly indicates that the shift in existing cropping-practices and cultivation of millet-based crops began at ~4,200±100 yrs BP aridity event in the peripheral (arid) zone of Indus civilization. Similar patterns were also observed at Kanmer [3] during the late phase of Mature Harappan era in the same ecological zone.

Botanical evidences suggest that the ~4,200yrs BP arid event may have forced a shift in agriculture towards millet type (also C-4 type) crops at Khirsara and other human-occupied sites in the region. Archaeological evidence from phase 1B also reveals a decline in technological sophistication and possibly also the lower economic status of the resident population. The shift towards millet-based food habits may have been an adaptative step in response to prevalent arid climate, which prevented a total collapse of civilization during this extreme event. However, the lower nutritional and caloric efficacy of millets in comparison to barley-wheat based crops may have contributed to a decline and eventual abandonment of the area [47].

### Concluding remarks

Our study from the Indus archaeological site at Khirsara (Gujarat, western India) provides compelling evidence for a major change in cropping pattern from barley- based to millet-based crops at ~4,200 yrs BP, suggesting that it was probably the first ever agricultural (human) response to prevailing monsoonal dryness. The combined macrobotanical, carbon isotopic and ceramic data collectively point to human sustenance at and after ~4,200 yrs BP dry event but with a significantly reduced economic status. Our findings are relevant to assessment of climate-culture relationships in the context of abrupt and extreme monsoonal change in the semi-arid and arid regions of India in the future [48, 49]. In the current, anthropogenically forced climate change scenario, several regions of India are being projected to go dryer or suffer from a significant change in rainfall pattern [5]. Agricultural produce of these regions must therefore be carefully monitored [49]. Expanding irrigation networks and altering cropping patterns could be considered by policy makers in identified vulnerable regions. More well-dated and quantified multi-proxy palaeo records from archaeological sites of northwestern India, focusing especially on past water-plant relationships, could greatly help in developing pragmatic socio-economic models in the wake of increasing warming, monsoonal dryness, and change in rainfall patterns.

## Supporting information

S1 Fig The contour map of the excavation plan at Khirsara archaeological site (Kachchh, Gujarat India). Black arrows show locations of major trenches AF-35 and Z-38 used for recovering palaeo- subsistence patterns and chronostratigraphy of depth scale.
(TIFF)
