# Supplementary figures and images for: Altered cropping pattern and cultural continuation with declined prosperity following abrupt and extreme arid event at ~4,200 yrs BP: Evidence from an Indus archaeological site Khirsara, Gujarat, western India
Source: PLoS One. 2017 Oct 6;12(10):e0185684. doi: 10.1371/journal.pone.0185684 (PMC5630146; doi:10.1371/journal.pone.0185684)

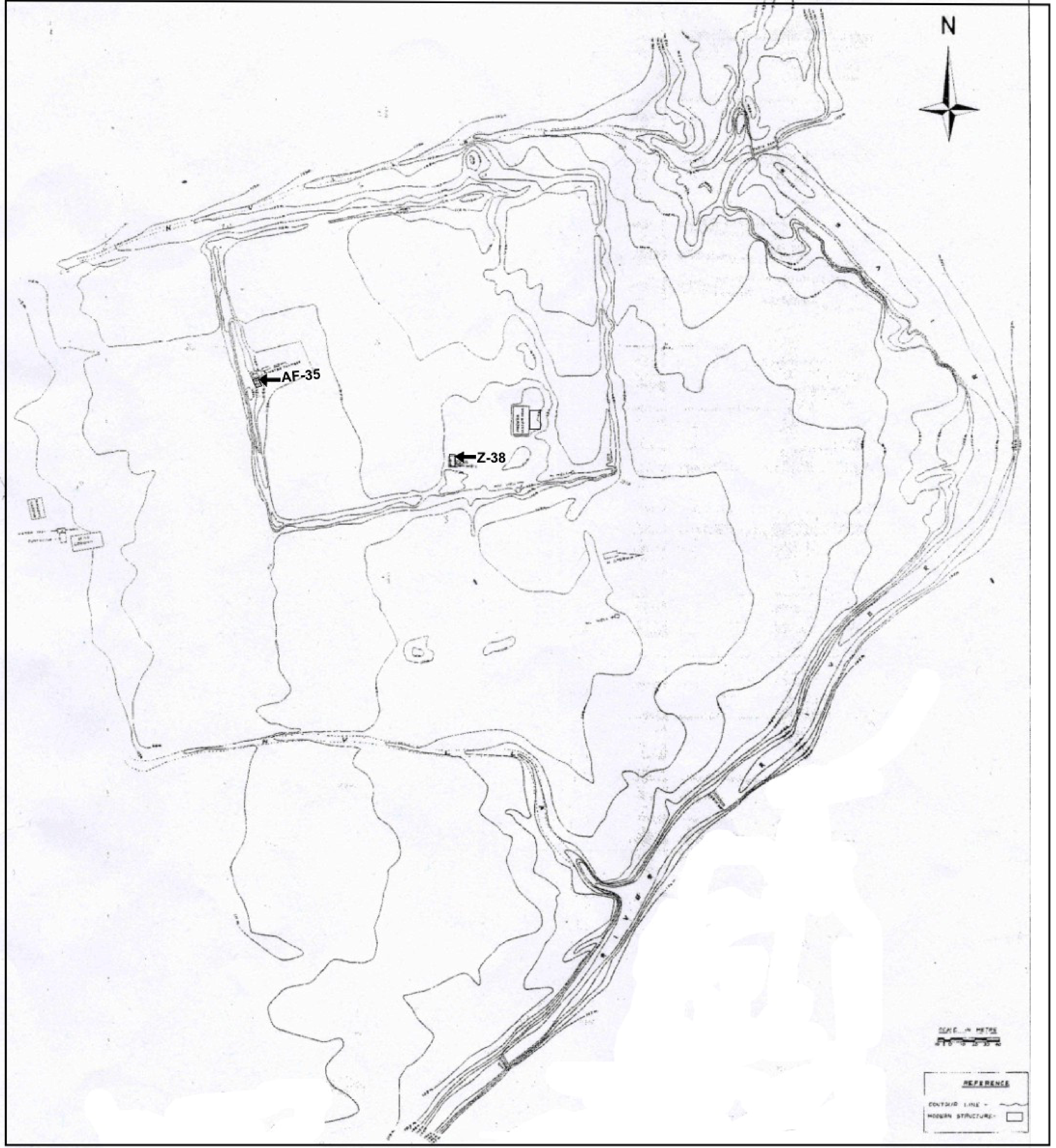

Supplement: S1 Fig — Black arrows show locations of major trenches AF-35 and Z-38 used for recovering palaeo- subsistence patterns and chronostratigraphy of depth scale. (TIFF) [file pone.0185684.s001.tiff]
